# Tuberous Sclerosis Complex as a Social Determinant of Health, Alongside Its Psychiatric Comorbidities: A Case Report

**DOI:** 10.7759/cureus.54956

**Published:** 2024-02-26

**Authors:** Hitesh P Rai, Cameron Sandefur

**Affiliations:** 1 Research, Edward Via College of Osteopathic Medicine, Monroe, USA; 2 Internal Medicine, Willis-Knighton Pierremont Hospitalist Group, Shreveport, USA

**Keywords:** internal medicine, public health, primary care, tuberous sclerosis, psychiatry

## Abstract

Tuberous sclerosis complex (TSC) is a neurocutaneous disease that manifests across multiple body systems. While there are substantial guidelines and protocols for managing the physical presentation of the disease, managing the psychosocial factors and the adverse effects as a social determinant of health is complex and unclear. This study discusses a patient with TSC who was hospitalized for pneumonia and how both her psychiatric and somatic symptoms were managed. Here we present the case of a 38-year-old Caucasian female with shortness of breath and generalized weakness. She had a past medical history of TSC and pneumothorax. This patient presented to the emergency department agitated and initially combative with her care team. Ultimately, she was administered dexmedetomidine to reduce her agitation. Here we suggest that this patient’s agitation and other psychiatric symptoms are intimately related to her diagnosis of TSC. Because of the heavy burden of TSC on the patient’s life, the patient’s aggressive nature could be an act of displacement of feelings from her medical complications onto her interactions with others. The patient understood that her complications hindered her ability to function as a healthy 38-year-old and perform activities of daily living without severe exhaustion. Just as her condition and its secondary complications hindered her body, feelings of anger and despair hindered her ability to appropriately interact and socialize with others. TSC is a debilitating condition that targets the body and mind. While much research has gone into treating each somatic system it may affect, there is a disconnect between the psychiatric aftermath and the toll that such a condition's psychiatric comorbidities may take on its patients.

## Introduction

Tuberous sclerosis complex (TSC) is a neurocutaneous disorder that manifests across multiple organ systems [1]. TSC is caused by loss-of-function mutations in one of two genes: TSC1 or TSC2, encoding hamartin and tuberin, respectively [1]. TSC is an uncommon disorder with an annual incidence rate of approximately one in 17,785 live births annually [2]. It is estimated to affect one in 50,000 individuals globally [3]. TSC presents through a variety of manifestations as it is a disorder of cellular proliferation and migration producing hamartomas affecting the skin, brain, heart, kidneys, lungs, or eyes in a manner that can vary across patients and time [4].

The vast phenotypic variability of TSC is responsible for its associated clinical pleiotropy [4]. Clinical presentation is often characterized by skin lesions, including ash leaf spots, seizures, and cellular overgrowths, either in the heart, kidneys, brain, or lungs [5,6]. Common presentations of TSC also include TSC-associated neuropsychiatric disorders, such as autism spectrum disorder and cognitive disability [1]. However, while 90% of patients with TSC may experience a range of behavioral, psychiatric, intellectual, academic, neuropsychologic, or psychosocial difficulties, only 20% of patients receive evaluation and treatment for these issues [7]. Nevertheless, there is adequate information on the diagnosis, management, and treatment of the physical manifestations of TSC.

The International TSC Consensus Conference in 2021 yielded a guideline for diagnosing TSC, which incorporated genetic testing alongside clinical criteria for diagnosis. The summarized diagnostic criteria discussed below provide a clearer picture of what is sufficient to make either a possible or definite diagnosis of TSC.

Genetic diagnostic criteria: Positive identification of either a TSC1 or TSC2 pathogenic DNA mutation from normal tissue via conventional genetic testing is sufficient to make a definite diagnosis [8]. An absence of positive identification of mutation via conventional genetic testing or a normal result does not exclude TSC or have any effect on the use of clinical diagnostic criteria to diagnose TSC [8].

Clinical diagnostic criteria: Subdivided into major and minor features. Major features include hypomelanotic macules (>3, at least 5 mm in diameter), angiofibromas (>3) or fibrous cephalic plaque, ungual fibromas (>2), shagreen patches, multiple retinal hamartomas, cortical dysplasias, subependymal nodules, subependymal giant cell astrocytoma, cardiac rhabdomyoma, lymphangioleiomyomatosis (LAM), and angiomyolipomas (>2) [8]. Minor features include “confetti” skin lesions, dental enamel pits (>3), intraoral fibromas (>2), retinal achromic patches, multiple renal cysts, nonrenal hamartomas, and sclerotic bone lesions [8].

A combination of LAM and angiomyolipomas without other features does not meet the criteria for a definite diagnosis, which consists of two major features or one major feature with >2 minor features. A possible diagnosis consists of either one major feature or >2 minor features [8].

Management and treatment of TSC in individuals is largely supportive, with surgical treatment as the conventional therapy for pathological hamartomas [9,10]. However, an increased understanding of the genetic cause of the disease and the underlying mammalian target of rapamycin (mTOR) pathway dysregulation led to clinical trials exploring the use of mTOR inhibitors, such as everolimus and sirolimus [10]. Furthermore, vigabatrin has been indicated as a first-line monotherapy treatment for TSC-related spasms or focal seizures in children aged one and younger [11]. Some studies have suggested the use of cannabidiol or incorporating a ketogenic diet to manage TSC-related epilepsy as well [12]. Here, we present the case of a patient with TSC and discuss the clinical symptoms and psychological impact of this condition. Through the presentation of this case, we hope to further educate healthcare providers on the importance of screening patients with TSC for psychiatric conditions and implementing an interdisciplinary treatment approach to assist patients in managing the high burden of illness associated with TSC.

## Case presentation

A 38-year-old Caucasian female presented to the emergency department with a chief complaint of shortness of breath and generalized weakness. After she was stabilized, she was transferred out of the ICU. This patient had concerns regarding her three-day history of shortness of breath, generalized weakness, bilateral lower extremity, and abdominal swelling. The patient states that these symptoms began shortly after her recent heroin and methamphetamine intravenous drug use. Her past medical history included tuberous sclerosis and pneumothorax. She denied any history of past surgeries, medications, or any knowledge of family history and reported alcohol consumption, crack/cocaine use, and methamphetamine and heroin intravenous drug use. The patient also reported that she had no known drug allergies.

**Figure 1:**
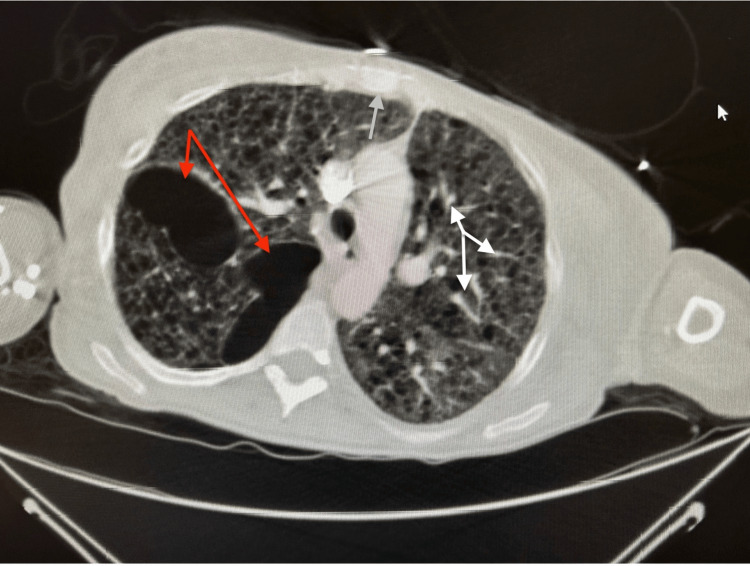
Chest CT reveals LAM, pulmonary infiltrates, and thoracic wall soft tissue edema CT: Computed tomography; LAM: Lymphangioleiomyomatosis; Red arrows: LAM; White arrows: Pulmonary infiltrates; Grey arrow: Thoracic wall soft tissue edema

**Figure 2:**
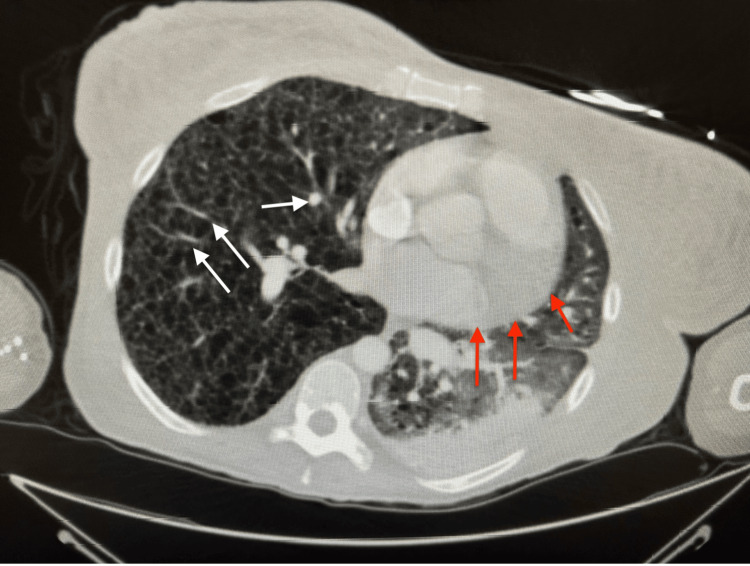
Chest CT confirms left lower lobe PNA and multiple sclerotic foci CT: Computed tomography; L: Left; PNA: Pneumonia; Red arrows: Lobar PNA; White arrows: Sclerotic foci

**Figure 3:**
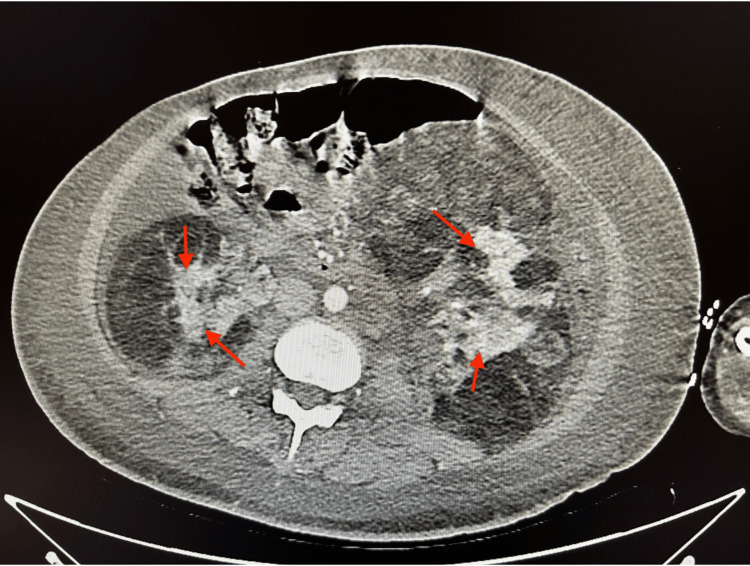
Abdominal CT visualizes multiple renal angiomyolipomas bilaterally CT: Computed tomography; Red arrows: Renal angiomyolipomas

On physical examination in the ICU, the patient was moderately distressed and short-tempered, with lower extremity edema present bilaterally. Moreover, hypomelanotic macules were visualized on the patient. The patient was previously agitated before rounding and was administered dexmedetomidine episodically, which successfully reduced her agitation. Her vitals revealed a blood pressure reading of 105/73 mmHg, pulse of 60 bpm, respiratory rate of 12 breaths per minute, temperature reading of 97.20 F, and an oxygen saturation reading of 97% on nasal cannula at a flow rate of four liters per minute. The patient was hypercapnic with an arterial blood gas carbon dioxide reading of 56.7, and a previously ordered urinalysis indicated a urinary tract infection (UTI), with a urine culture providing a positive result for E. coli. Imaging modalities revealed left lower lobe pneumonia on X-ray, and a CT of the chest and abdomen also visualized her pneumonia and revealed LAM cysts within her lungs, multiple osseous sclerotic foci, renal angiomyolipomas bilaterally, and thoracic wall soft tissue edema (Figures 1-3).

The primary assessment of the patient included diagnoses of community-acquired pneumonia, sepsis, acute respiratory failure with hypoxia and hypercapnia, UTI, anemia, acute agitation, tuberous sclerosis, and a history of intravenous drug use. The plan included administering cefepime, doxycycline, and methylprednisolone for pneumonia, sepsis, and UTI. A bilevel-positive airway pressure ventilator was ordered to manage acute respiratory failure, as well as a nebulizer, which was also used to treat the patient’s pneumonia. Episodes of acute agitation were managed with dexmedetomidine, and the patient’s anemia was managed with iron sucrose. The patient was also counseled on the risks associated with intravenous drug use and was instructed to follow up with her outpatient pulmonologist upon discharge. Once the pneumonia and UTI had cleared, a six-minute walk test was ordered to measure the level of the patient’s lung impairment. The patient’s oxygen saturation dropped below 88% to a low 70% saturation during the six-minute walk test. Thus, she was provided with supplemental oxygen. Based on the patient's presentation, lack of a support system, and her social worker's recommendation, the patient's discharge plan was adjusted. Ultimately, the patient was discharged to long-term acute care after her hospitalization.

## Discussion

This patient’s acute manifestations of her TSC were managed and resolved appropriately. However, her intravenous drug use, agitation toward healthcare staff, and volatile temperament may be manifestations of a deeper issue. It appears that these are often overlooked behavioral manifestations, which may be associated with the physiological disease process. While research on TSC has primarily focused on the physical manifestations of the disease, one study on the burden of illness and quality of life in patients with TSC focused on the psychosocial aspects: the study detailed that 42.1% of patients reported experiencing negative progress in their education or career because of TSC, and 76.5% of caregivers indicated that TSC affected family life alongside social and working relationships [13]. Furthermore, 35% of patients with TSC reported routine pain and/or discomfort, and 43.4% reported anxiety and/or depression across all ages and levels of disease [13]. Thus, TSC serves as an adverse social determinant of health. Furthermore, another study including 241 patients with TSC revealed that 26% had been diagnosed with a mood disorder, 28% with an anxiety disorder, 21% with adjustment disorders, 21% with attention-deficit hyperactivity disorder, and 42% with unspecified mental health disorders [14]. The overall rate of 66% of patients in the study’s population that qualified for at least one psychiatric symptom suggests a substantial psychiatric comorbidity in TSC [14]. Social determinants of health alongside psychiatric comorbidities should be considered when discussing this patient’s decisions and behaviors, as they could potentially serve as confounding variables.

This case discussed a patient with TSC and the course of her hospitalization, highlighting the associated psychiatric findings and psychosocial impacts of the disease on her entire treatment. Future studies may also benefit from placing an increased focus on interdisciplinary care and mental health screening in patients with TSC. This case serves to bring greater attention to the comorbidity of psychiatric conditions associated with those affected by TSC, as well as the adverse effects it has as a social determinant of health.

## Conclusions

The findings in this patient’s hospitalization, while not atypical in physical manifestation from other cases of TSC, highlight the factors that may have exacerbated her condition. The patient’s temperament, lifestyle choices, and physical condition all contribute to her overall health. Surely her presentation bridges the gap between mind and body. Medical practice may benefit tremendously from a better understanding of the given burden of illness and how it reciprocally plays into further exacerbation of the disease process. In the case of this patient and her TSC, more research may provide a clearer picture of that exact phenomenon.
